# Curcumin attenuates autoimmunity and renal injury in an experimental model of systemic lupus erythematosus

**DOI:** 10.14814/phy2.14501

**Published:** 2020-07-11

**Authors:** Elena L. Dent, Erin B. Taylor, Hannah R. Turbeville, Michael J. Ryan

**Affiliations:** ^1^ Department of Physiology & Biophysics University of Mississippi Medical Center Jackson MS USA; ^2^ Department of Pharmacology and Toxicology University of Mississippi Medical Center Jackson Mississippi USA; ^3^ GV (Sonny) Montgomery Veterans Affairs Medical Center Jackson Mississippi USA

**Keywords:** autoantibodies, autoimmunity, curcumin, hypertension, systemic lupus erythematosus

## Abstract

Systemic lupus erythematosus (SLE) is an autoimmune disorder with prevalent hypertension and renal disease. To avoid side effects of immunosuppressive drugs, alternative therapies are needed. Curcumin has been used in Eastern medicine for its anti‐inflammatory and antioxidant properties. This study tested whether oral curcumin administration attenuates autoimmunity and renal injury during SLE. Female NZBWF1 (model of SLE) and NZW/LacJ (control) mice were administered curcumin (500 mg kg^‐1^ day^‐1^, oral gavage) for 14 days in two separate groups beginning at either 26 or 32 weeks of age. Body weight and composition were monitored throughout the study. Immune activity was assessed by spleen weight, circulating dsDNA autoantibodies, and B lymphocytes. Renal injury (albumin excretion, glomerulosclerosis, blood urea nitrogen (BUN)) was measured as a hemodynamic function (glomerular filtration rate (GFR), mean arterial pressure (MAP)) in conscious mice. Body weight and composition were maintained in curcumin‐treated SLE mice, but decreased in vehicle‐treated SLE mice. Curcumin‐treated SLE mice had lower spleen weight and renal injury (glomerulosclerosis) compared to vehicle‐treated SLE mice when treatment started at 26 weeks of age. When curcumin treatment started at 32 weeks of age, renal injury (glomerulosclerosis, BUN) was reduced in SLE mice compared to vehicle‐treated SLE mice. GFR was reduced, and MAP was increased in vehicle‐treated SLE mice compared to controls; however, these were not improved with curcumin. No significant changes were observed in curcumin‐treated control mice. These data suggest that curcumin modulates autoimmune activity and may lessen renal injury in female mice with SLE.

## Graphical abstract

Patients with autoimmune diseases like systemic lupus erythematosus take medications that have untoward side effects. This study tests whether a dietary intervention with curcumin attenuates the development of renal disease during SLE.

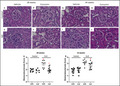

## 1 INTRODUCTION

Systemic lupus erythematosus (SLE) is a prototypical autoimmune disease characterized by the presence of circulating autoantibodies (commonly to nuclear antigens), systemic and localized inflammation, renal injury, hypertension, and cardiovascular disease. This multisystem disease has a strong female predilection with an estimated female‐to‐male ratio of 9:1 (Aggarwal, Sundaram, Malani, and Ichikawa, 2007; Ahmed, Khan, & Mirzaei, 2019; Al‐Herz, Ensworth, Shojania, & Esdaile, 2003), is often diagnosed in women during their childbearing years (Ahmed et al., 2019; Al‐Herz et al., 2003), has a high prevalence of hypertension (Anders and Weening, 2013), a high incidence of resistant hypertension (Andrews et al., 1978), and a high incidence of renal involvement (Aparicio‐Trejo et al., 2017; Bassi et al., 2015; Batlle‐Gualda, Martinez, Guerra, & Pascual, 1997; Baziar & Parohan, 2020).

Medical treatment for SLE involves a combination of induction and maintenance immunosuppressive therapy. For example, a patient may be prescribed a combination of intravenous (i.v.) cyclophosphamide and oral steroids as an aggressive induction therapy to suppress an active disease flare. Patients who do not have active disease are prescribed immunosuppressant therapy in a maintenance regimen (Boumpas et al., 1995) to reduce the risk of a new flare and to preserve renal function. Mycophenolate mofetil and azathioprine are often prescribed in order to keep, or maintain, the disease in remission. These therapies target a wide range of cells including T and B lymphocytes, leukocytes, and rapidly dividing cells. By depleting these cells of the immune system, increased risk of infection is a serious side effect (Budman & Steinberg, 1976) along with the potential for poor wound healing, nausea, vomiting, infertility, osteoporosis, and general malaise, leaving patients and providers searching for less toxic adjunctive or alternative therapies.

Curcumin, most commonly consumed as the spice turmeric, is derived from the plant *Curcuma longa*, (Batlle‐Gualda et al., 1997) and is a known polyphenolic compound (Burnett, Ravel, & Descotes, 2004) traditionally used in South East Asian and Indian medicine for its anti‐inflammatory and antioxidant properties (Chung et al., 2007). Curcumin therapy is a potentially attractive adjuvant, or alternative, therapy for patients with autoimmune diseases as it is generally recognized as safe, has been reported to attenuate inflammation, suppress autoimmune activity (Corna et al., 1997; Cozzani, Drosera, Gasparini, & Parodi, 2014), upregulate anti‐inflammatory cytokines (Davidson et al., 2015), and protect the kidneys in 5/6 nephrectomy rats (Dei Cas & Ghidoni, 2019; Dent, Taylor, Sasser, & Ryan, 2020; Eriguchi et al., 2018). Curcumin has been shown to increase intestinal alkaline phosphatase expression to counteract the effects of inflammation on gut permeability that is associated with chronic kidney disease and other inflammatory disorders, and therefore reduce inflammatory biomolecules in the circulation of LDL receptor knockout mice (Fiehn et al., 2003). Curcumin has also been touted as cardioprotective by reducing systolic blood pressure in humans (Frese‐Schaper, Zbaeren, Gugger, Monestier, and Frese, 2010) and experimental models via downregulation of the angiotensin 1 receptor expression in arteries (Gandelman et al., 2020), reducing low‐density lipoprotein levels and oxidative stress, and promoting vasorelaxation in porcine coronary arterial ring segments via nitric oxide release (Ghayur & Margetts, 2013; Ghosh, Gehr, & Ghosh, 2014). In a mouse model of Alzheimer's disease, curcumin mixed in chow for 12 weeks lowered neuroinflammation and consequently neurodegeneration (Gilbert & Ryan, 2011). The impact of curcumin on the development of SLE and associated cardiovascular and renal risk factors is incompletely understood. Therefore, the present study examined the hypothesis that the administration of curcumin will lessen autoimmune activity and renal injury in female mice with SLE.

## 2 METHODS

### 2.1 Animal model

Female NZBWF1 mice, an established murine model of lupus (Gilbert and Ryan, 2014), and female control (NZW/LacJ) mice were obtained from The Jackson Laboratories (Bar Harbor, ME). Distinct groups of control and SLE mice were studied at ages (26–28, and 32–34 weeks of age) with a total cohort of 46 (control) and 45 (SLE) mice. This was done to assess whether the potential efficacy of curcumin was partly dependent upon the age of the mice at the start of the treatment. The data from each age comprised of mice from multiple litters across 1‐year period. Mice were housed with access to normal chow and water ad libitum and kept on a 12‐hr light/dark cycle at room temperatures between 21 and 23°C. At the end of the study, animals were killed via isoflurane and cervical dislocation and tissues were collected for analysis. All studies were approved by the University of Mississippi Medical Center (UMMC) Institutional Animal Care and Use Committee (IACUC) and were in accordance with National Institutes of Health (NIH) Guide for the Care and Use of Laboratory Animals.

### 2.2 Diet composition

Mice received standard chow, Envigo Teklad 8640, containing 22% protein, 9% fat, and 40.6% carbohydrate, ad libitum. SLE and control mice were supplemented with high‐purity curcumin obtained from Sigma‐Aldrich (C7727‐500 mg). Mice were administered 0.5 ml of corn oil (vehicle) or curcumin (500 mg kg^‐1^ day^‐1^) mixed in 0.5 ml of corn oil by oral gavage for 14 days beginning at 26 or 32 weeks of age.

### 2.3 Body composition

The percentage of fat and lean tissue in curcumin‐treated SLE and control mice was determined at 32, 33, and 34 weeks of age using echo MRI as previously described by our laboratory (Hadi, Pourmasoumi, Ghaedi, & Sahebkar, 2019). Mice were weighed daily during the study.

### 2.4 Autoantibody production

Plasma samples were taken at baseline (26 or 32 weeks) before the start of curcumin or vehicle administration, and again at the end of the study (28 and 34 weeks). The plasma was analyzed for anti‐dsDNA IgG autoantibodies using a commercial ELISA (Alpha Diagnostic International) per manufacturer's instructions and as described previously by our laboratory (Han, Zhuang, Shumyak, Yang, & Reeves, 2015; Hashish & Elgaml, 2016). Results are presented in kU/ml.

### 2.5 Flow cytometry

Blood samples were collected from the retro‐orbital plexus of control and SLE mice at baseline and at the end of the study for flow cytometry analysis of CD45R+ B cells. Cells were prepared and analyzed as previously described by our laboratory (Jacob et al., 2013), stained with anti‐CD45R‐APC (clone RA3‐6B2) obtained from BD Biosciences, catalog number 557,683, and analyzed using a Gallios flow cytometer (Becton Dickinson) at the UMMC flow cytometry core facility.

### 2.6 Albuminuria

Daily urinary albumin excretion rate (mg/day) was measured by ELISA (Alpha Diagnostics International) using 24‐hr urine samples, as previously described by our laboratory (Hashish & Elgaml, 2016; Kim, 2014) at baseline and at the end of the study.

### 2.7 Glomerulosclerosis

Glomerulosclerosis was assessed using a hematoxylin and eosin staining protocol. Approximately 30 glomeruli per mouse were scored blinded using a scale of 0–4; 0 = no sclerosis, 1 = 0%–25% of glomeruli have sclerosis, 2 = 25%–50% of glomeruli have sclerosis, 3 = 50%–75% of glomeruli have sclerosis, and 4 = 75%–100% of glomeruli have sclerosis. A total glomerular score was tabulated by adding the number of 0’s, 1’s, 2’s, 3’s, and 4’s multiplied by the number of glomeruli with that scale score and divided by the total number of glomeruli scored.

### 2.8 Blood urea nitrogen (BUN)

Plasma BUN was assessed at baseline and at the end of the study by a Vet Axcel Chemistry Analyzer (Alfa Wasserman, Diagnostic Technologies, LLC) using 40 μl of plasma.

### 2.9 Blood pressure and glomerular filtration rate measurement

A subset of mice was instrumented with jugular vein and carotid artery catheters under isoflurane anesthesia for measurement of blood pressure and glomerular filtration rate (GFR) in the same conscious animals. Mice were allowed to recover overnight before recording blood pressure in conscious, freely moving mice for 90 min using an 8‐channel PowerLab/16SP (ADInstruments) blood pressure transducer. Data were recorded using Chart5 for Windows (ADInstruments).

Glomerular filtration rate was measured in conscious, restrained mice by indwelling jugular vein catheterization and constant infusion of fluorescein isothiocyanate (FITC)‐inulin using a Harvard pump (0.004 ml/min). Infusions were performed immediately after blood pressure recording. The carotid catheter was used to collect plasma samples at 4‐, 4.5‐, and 5‐hr time points to ensure steady state was reached (defined as two consecutive samples with a FITC‐inulin concentration within 10% of each other). At the end of the infusion protocol, 10 μl of plasma from each time point was transferred to a 96‐well clear bottom black plate and analyzed for fluorescence at 485 nm excitation and 530 nm emission. Plate readings (concentration) were used to calculate GFR using the following formula: $\text{GFR}=\frac{\left[\text{Infusate}\right]\times \left[\text{Rate}\;\text{of}\;\text{Infusion}\right]}{\left[\text{Plasma}\right]}$. Results were corrected for total kidney weight.

### 2.10 Statistical analysis

**Figure 1:**
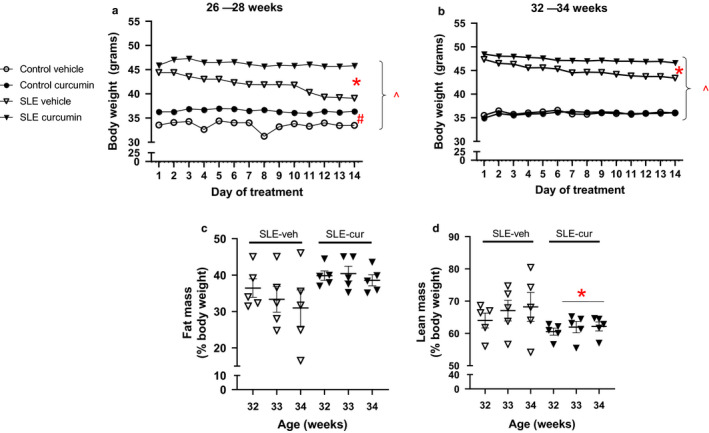
(a and b) Body weight is preserved in curcumin‐treated SLE mice at 34 weeks of age. Collectively, body weight is significantly higher in SLE mice compared to control mice at 28 and 34 weeks of age, ^*p* < .001, two‐way ANOVA. Body weight is maintained in curcumin‐treated SLE compared to vehicle‐treated SLE mice at 28 and 34 weeks of age,*p < .001, two‐way ANOVA. Body weight is lower in vehicle‐treated control mice compared to curcumin‐treated control mice at 28 weeks of age, ^#^ *p* < .001, two‐way ANOVA, and is maintained in curcumin‐ and vehicle‐treated control mice at 34 weeks of age. (c and d) Fat and lean mass are not significantly different between curcumin‐treated SLE mice compared to vehicle‐treated SLE mice at 34 weeks of age; however, there was an effect of treatment (vehicle versus curcumin) on lean mass, *p = .0357, two‐way ANOVA (d)

Data are presented as standard error of mean unless stated otherwise. Statistical outliers, identified as greater than two standard deviations higher than the mean, were omitted. Statistical analyses were performed using GraphPad Prism 6 (GraphPad Software). A two‐way ANOVA with multiple comparisons (Tukey for all figures except Figure 1c and d, which used the Sidak's multiple comparison test) was used to determine differences within groups. Values were considered statistically significant at *p* = <.05.

## 3 RESULTS

### 3.1 Body composition

Systemic lupus erythematosus mice had statistically greater body weight compared to control mice throughout the 28 and 34 week studies, *p* = <.0001. Curcumin‐treated control and SLE mice had no change in body weight throughout the study. Vehicle‐treated SLE mice had significantly lower body weight compared to age‐matched curcumin‐treated SLE mice at 28 and 34 weeks of age, *p* = <.0001. Vehicle‐treated control mice had lower body weight compared to curcumin‐treated control mice at 28 weeks of age, *p* = <.0001. No differences were seen between control vehicle‐ or treated mice at 34 weeks of age (Figure 1a and b). SLE mice had statistically greater fat mass compared to control mice which is consistent with previous studies from our laboratory (Kim et al., 2017). In the current study, we examined body composition in vehicle‐ and curcumin‐treated SLE mice only during the 32‐ to 34‐week study because past studies show that NZBWF1 mice are more likely to be cachectic at this age. Fat mass does not change in vehicle‐ or curcumin‐treated mice, whereas lean mass is preserved in curcumin‐treated SLE mice (treatment effect) (Figure 1c and d).

### 3.2 Spleen weight

**Figure 2:**
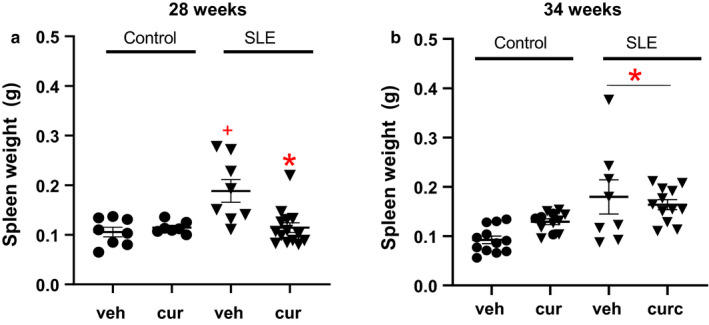
(a) Spleen weight was significantly higher in SLE vehicle‐treated mice compared with age‐matched vehicle‐ and curcumin‐treated control mice, +*p* < .007, two‐way ANOVA. Spleen weight is decreased in curcumin‐treated SLE mice at 28 weeks of age compared to age‐matched vehicle‐treated SLE mice,*p < .002, two‐way ANOVA. (b) Spleen weight is higher in SLE at 34 weeks of age compared to control mice, *p < .005, two‐way ANOVA. Spleen weight is unchanged in vehicle‐ or curcumin‐treated control and SLE mice at 34 weeks of age

Consistent with previously published studies, spleen weight was higher in vehicle‐treated SLE mice compared to control mice at both time points, suggesting higher immune activity in SLE mice (Lee, Kim, Lee, Chung, & Bae, 2013). There was a significant group effect on spleen weight (SLE versus Control) at both 28 and 34 weeks of age. Multiple comparisons testing revealed that spleen weight was significantly lower in curcumin‐treated SLE mice at 28 weeks of age (Figure 2 and b) compared to age‐matched vehicle‐treated SLE mice. However, this was not observed in mice at 34 weeks of age.

### 3.3 Autoantibodies

**Figure 3:**
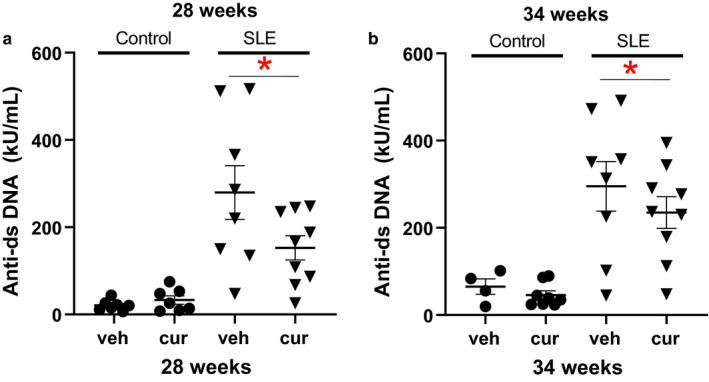
(a) Anti‐dsDNA IgG is significantly higher in SLE mice compared to controls (*p < .001 versus control, two‐way ANOVA, group effect) at 28 weeks of age. (b) Anti‐dsDNA IgG is significantly higher in SLE mice compared to controls (two‐way ANOVA, group effect) at 34 weeks of age

Anti‐dsDNA IgG autoantibodies are used for evaluation of disease activity and diagnosis (Li et al., 2019; Mathis et al., 2013) of SLE in humans. Compared to control mice, SLE mice had higher levels of anti‐dsDNA IgG autoantibodies (Figure 3a and b, two‐way ANOVA, group effect). Curcumin‐treated SLE and control mice had unchanged anti‐dsDNA IgG levels.

### 3.4 Flow cytometry

**Figure 4:**
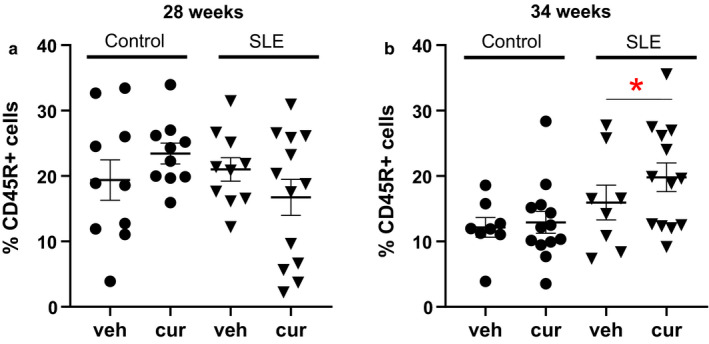
(a) The percentage of circulating CD45R + B cells is not different between SLE and control mice, either vehicle or treated, at 28 weeks of age. (b) At 34 weeks of age, circulating CD45R + B cells are significantly increased compared with age‐matched controls, *p = .0166, two‐way ANOVA, group effect

The percentage of circulating CD45R+ B lymphocytes was assessed using flow cytometry. Although there was a significant increase in CD45R+ B lymphocytes at 34 weeks (SLE versus Control, two‐way ANOVA, group effect), the percentage of CD45R+ B cells was not changed in curcumin‐treated control and SLE mice (Figure 4a and b).

### 3.5 Albuminuria

**Figure 5:**
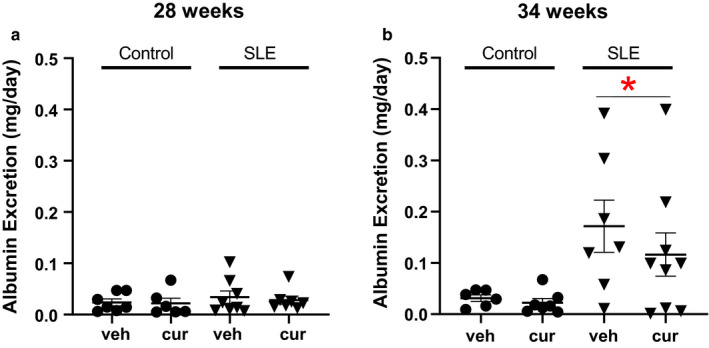
(a) Albumin excretion was unchanged in vehicle or treated control and SLE mice at 28 weeks of age. (b) Albumin excretion is higher in SLE mice compared to age‐matched controls (*p = .0040 versus control, two‐way ANOVA, group effect)

Albuminuria is a common, albeit variable, finding in patients with SLE (Aparicio‐Trejo et al., 2017; Mathis et al., 2012; Mayes et al., 2003), and in female NZBWF1 mice (Kim et al., 2014). Systemic lupus erythematosus mice had significantly higher albumin excretion compared to control mice at 34 weeks of age measured by ELISA (two‐way ANOVA, group effect). Two outliers were identified as being greater than 2 standard deviations higher than the mean, 1 in the vehicle‐treated SLE group, and 1 in the curcumin‐treated SLE group, at 34 weeks of age and were omitted from the analysis. There was no significant difference between vehicle and curcumin‐treated SLE mice. Control mice did not develop albuminuria at either time point during this study (Figure 5a and b).

### 3.6 Glomerulosclerosis

**Figure 6:**
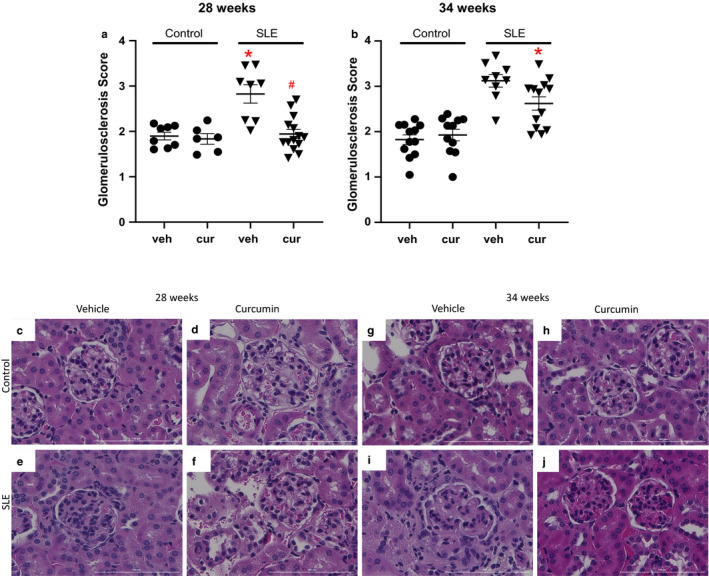
(a) Glomerulosclerosis is significantly increased in SLE mice at 28 weeks of age compared to age‐matched vehicle‐ and curcumin‐treated control mice (*p < .001 versus control, two‐way ANOVA, group effect). Curcumin‐treated SLE mice had less glomerulosclerosis compared to vehicle‐treated SLE mice at 28 weeks of age (^#^ *p* < .001). (b) Glomerulosclerosis is significantly increased at 34 weeks of age in SLE mice compared to age‐matched control mice (*p < .001 versus control, two‐way ANOVA, group effect). Glomerulosclerosis is significantly decreased at 34 weeks of age in curcumin‐treated SLE mice compared to age‐matched vehicle treated SLE mice,*p = .05. (c–j) Representative pictures of glomerulosclerosis (40x) from paraffin‐embedded kidneys stained with hematoxylin and eosin

Glomerular scoring was assessed for vehicle‐ and curcumin‐treated control and SLE mice at 28 and 34 weeks of age. Consistent with previously published studies from our laboratory (Jacob et al., 2013; Mollazadeh et al., 2019), glomerular injury was not present in control mice while glomerular injury was present by 28 and 34 weeks of age in vehicle‐treated SLE mice (Figure 6, two‐way ANOVA, group effect). At both 28 and 34 weeks of age, curcumin‐treated SLE mice had less glomerular injury.

### 3.7 Blood urea nitrogen

**Figure 7:**
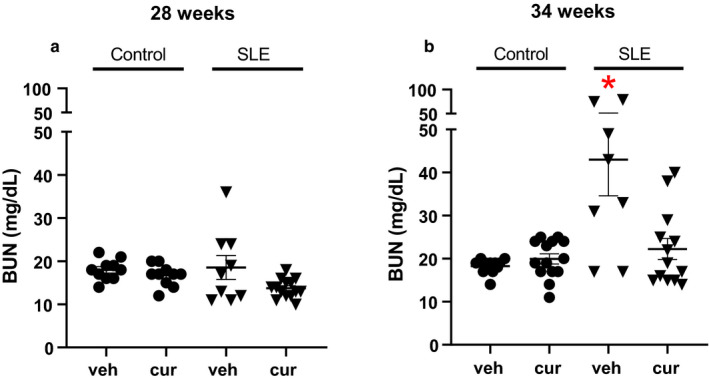
(a) Blood urea nitrogen (BUN) is not different between SLE and control mice at 28 weeks of age. (b) BUN is significantly increased in SLE mice compared to age‐matched control mice (*p < .001 versus control, two‐way ANOVA, group effect). Curcumin‐treated SLE mice have lower BUN compared to vehicle‐treated SLE mice. * *p* < .001 versus vehicle control, curcumin control, vehicle SLE

Plasma BUN levels are used clinically to assess renal function (Momtazi‐Borojeni et al., 2018). One outlier was identified as greater than 2 standard deviations higher than the mean in the vehicle‐treated SLE group at 28 weeks of age and was omitted from analysis. Vehicle‐treated SLE mice had higher levels of plasma BUN at 34 weeks of age compared to age‐matched control mice (two‐way ANOVA, group effect). Curcumin‐treated SLE mice had lower levels of plasma BUN at 34 weeks of age compared to vehicle‐treated SLE mice (Figure 7). Blood urea nitrogen remained unchanged in control mice across the study, consistent with previous published work from our laboratory, suggesting normal renal function (Mollazadeh et al., 2019).

### 3.8 Hemodynamics

**Figure 8:**
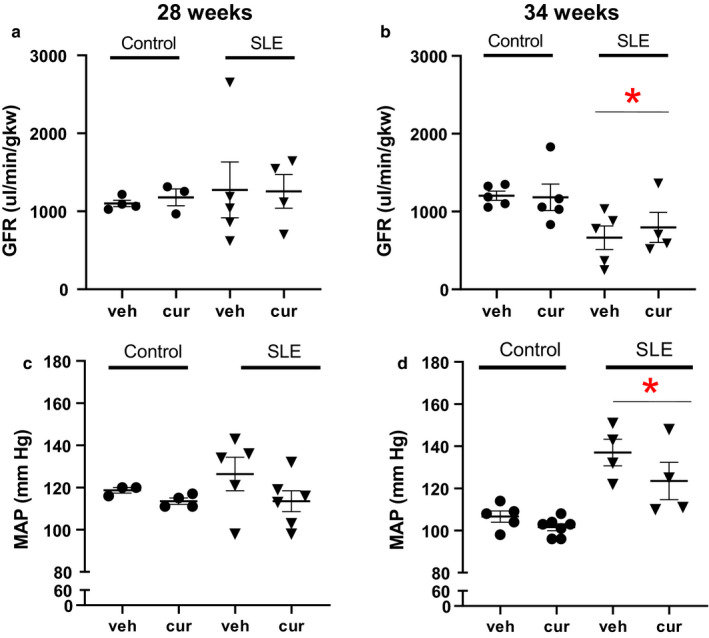
(a) Glomerular filtration rate (GFR) was not different between SLE and control mice at 28 weeks of age. (b) GFR was significantly lower in SLE mice compared to age‐matched controls (*p = .007 versus control, two‐way ANOVA, group effect). However, curcumin treatment did not affect GFR. (c) Mean arterial pressure (MAP) was not different between SLE and control mice at 28 weeks of age. (d) MAP is significantly higher in SLE mice compared to age‐matched control mice (*p < .001 versus control, two‐way ANOVA, group effect). However, curcumin treatment did not affect MAP

Blood pressure was not different in vehicle‐ and curcumin‐treated control and SLE mice at 28 weeks of age. However, at 34 weeks of age, MAP was significantly higher in SLE mice compared to age‐matched controls (two‐way ANOVA, group effect). Blood pressure was not altered in SLE mice treated with curcumin. Glomerular filtration rate is not different across the groups at 28 weeks of age in vehicle‐ and curcumin‐treated control and SLE mice. GFR was lower at 34 weeks of age in SLE mice compared to controls (two‐way ANOVA, group effect) consistent with our previously published work (Mollazadeh et al., 2019) (Figure 8). However, GFR was not preserved in mice treated with curcumin.

## 4 DISCUSSION

Systemic lupus erythematosus patients develop autoantibodies to nuclear components, renal impairment, and prevalent hypertension. Many of the immunosuppressive therapies prescribed cause severe side effects. Based on this, the current study explored the impact of an alternative nutrition‐related therapy on characteristics of disease activity, renal injury and function, and blood pressure. The hypothesis was tested that oral administration of curcumin will attenuate autoimmune activity and renal injury in female mice with SLE. This study advances the field by demonstrating that (a) SLE mice treated with curcumin are able to preserve their body weight, (b) spleen weight is lower in SLE mice treated with curcumin until 28 weeks of age, but not does affect circulating CD45R+ B cells or anti‐dsDNA IgG autoantibodies, (c) curcumin treatment attenuates glomerulosclerosis but does not affect albuminuria, and (d) curcumin, at the dose and duration in this study, does not alter systemic or renal hemodynamic function in female NZBWF1 SLE mice.

Body composition changes such as increases in visceral adiposity have been reported in human (Mousavi, Milajerdi, Varkaneh, Gorjipour, and Esmaillzadeh, 2020; Nakano et al., 1998) and experimental murine SLE cohorts (Neimann et al., 2006). Published data from our laboratory show that female mice with SLE have increased body weight and visceral adiposity relative to age‐matched control mice (Hadi et al., 2019). However, SLE mice experience a loss in body weight and fat mass by 34 weeks of age (Hadi et al., 2019). The data in the present study confirm the increased body weight and fat composition of our earlier studies, and show that curcumin‐treated SLE mice had preserved body weight at 34 weeks of age. This is consistent with an animal study in NZBWF1 mice treated with curcumin showing curcumin‐treated mice gained weight by 32 weeks of age (Batlle‐Gualda et al., 1997) but differs from a study in C57BL6 mice receiving curcumin supplementation in combination with α‐lipoic acid while on a high‐fat diet showed a reduction in weight and adiposity (Okudaira, Terada, & Okudaira, 1987). In human studies, a recent meta‐analysis of randomized controlled trials demonstrated that curcumin may positively affect body composition (weight and body mass index) and may reduce waist circumference in overweight individuals taking more than 1,000 mg of curcumin per day for at least 8 weeks (Panoulas et al., 2007; Panzhinskiy, Bashir, Bagchi, & Nair, 2019). One possible reason for the maintained body weight in SLE mice, as opposed to weight loss reported in other clinical conditions, is the impact of curcumin to suppress inflammation and immune system function, thus causing the mice to be more inclined to eat, and maintain their weight. The dose of curcumin administered to mice in this study is the equivalent of twice the amount given to humans (12 g/day) in recent clinical trials using curcumin (Pons‐Estel, Alarcón, Scofield, Reinlib, & Cooper, 2010). Although female mice with SLE weigh more than NZW/LacJ control mice on average, body weight decreases rapidly as the disease progresses in this model.

Spleen weight was used as a rudimentary indicator of immune activity given that NZBWF1 mice develop splenomegaly (Lee et al., 2013). The difference in spleen weight in curcumin‐treated 28‐week‐old SLE mice compared to vehicle suggests attenuation of active disease. SLE mice treated with curcumin beginning at 32 weeks of age did not demonstrate a difference in spleen weight, suggesting curcumin did not attenuate active disease at this time point. The lack of effectiveness is likely because the intervention was started after the disease had been active for several weeks.

Patients and experimental murine models with SLE commonly develop autoantibodies to nuclear components that correlate with disease activity (Ponticelli, Glassock, & Moroni, 2010; Rees, Doherty, Grainge, Lanyon, & Zhang, 2017). Anti‐dsDNA IgG is highly specific for SLE as it is found in approximately 90% of humans with SLE (Rees et al., 2017; Reveille, 2004). Our laboratory and others reported that anti‐dsDNA IgG autoantibodies progressively increase in SLE beginning as early as 20 weeks of age (Hadi et al., 2019; Rudofsky et al., 1984; Rudofsky & Lawrence, 1999; Ryan & McLemore, 2007) but remain relatively constant in age‐matched controls. In the current study, SLE mice exhibited higher circulating levels of anti‐dsDNA IgG autoantibodies compared to control mice at 28 and 34 weeks of age. Compared to vehicle‐treated SLE mice at 28 and 34 weeks of age, curcumin‐treated SLE mice did not have statistically significant differences in circulating anti‐dsDNA IgG autoantibodies. This contrasts with a study showing a decline in anti‐dsDNA IgG autoantibodies at 32 weeks of age in female NZBWF1 mice fed curcumin mixed in normal chow for 14 weeks (Batlle‐Gualda et al., 1997). The differences may be related to the fact that curcumin was only administered for 2 weeks, and the treatment was initiated after the onset of disease. To further evaluate immune activity, circulating CD45R+ B lymphocytes were assessed. Thirty‐four‐week‐old SLE mice displayed a greater percentage of circulating CD45R+ B cells compared to control mice; however, no differences were noted in curcumin‐treated control or SLE mice. These data suggest curcumin treatment, under these conditions, does not alter autoimmune activity via changes in anti‐dsDNA IgG autoantibodies or circulating CD45R+ B cells.

Renal involvement has been reported to affect approximately half or more of SLE patients (Rees et al., 2017; Ryan, McLemore, & Hendrix, 2006; Sabio et al., 2011). Markers of renal injury, such as albuminuria and glomerulosclerosis can be found in human and experimental murine data (Han et al., 2015; Jacob et al., 2013; Kim et al., 2014; Saxena, Mahajan, & Mohan, 2011; Seguro, Paupitz, Caparbo, Bonfa, & Pereira, 2018; Seki et al., 2019; Shaharir, Mustafar, Mohd, Mohd Said, & A. Gafor, 2015). The current study demonstrates SLE mice have increased albuminuria by 34 weeks of age compared to control mice, consistent with previously published data (Hashish & Elgaml, 2016; Song et al., 1998), and comparable to the biological variability of albumin excretion commonly observed in humans (Stucker & Ackermann, 2011). This is in contrast to a study by Lee et al. showing that curcumin‐treated NZBWF1 mice exhibit a significant decline in albuminuria by 32 weeks of age (Batlle‐Gualda et al., 1997). This discrepancy may also be due to the age at which the treatment started (18 weeks of age), and the duration of the treatment (14 weeks) compared to a duration of only 2 weeks of curcumin beginning at either 26 or 32 weeks of age in the present study. The shorter 2‐week treatment protocol may provide insight for the effectiveness of utilizing curcumin as a treatment, whereas the longer duration of curcumin administration from a younger age provides important insight as a preventative measure.

While curcumin administration did not appear to affect albumin excretion, curcumin‐treated SLE mice had less glomerulosclerosis at both 28 and 34 weeks of age when compared to age‐matched vehicle‐treated counterparts. This suggests curcumin has a beneficial impact on renal injury which has been previously reported in this murine model treated with curcumin (Batlle‐Gualda et al., 1997). The reasons behind the attenuated glomerulosclerosis in the curcumin‐treated animals are unclear. However, given that autoantibodies are unchanged, the renal protection may result from generalized anti‐inflammatory properties of curcumin. For example, curcumin can inhibit TGF‐β, an important stimulator of extracellular matrix (ECM) production that promotes fibrosis and glomerulosclerosis and has been reported to be elevated in chronic kidney disease (Sundaram et al., 2017). One study reported mice treated with curcumin had reduced ECM protein expression within the kidneys and reduced TGF‐β, leading to a reduction in glomerulosclerosis (Tapia et al., 2013).

Albuminuria may arise from a complex interplay of inflammation and glomerular filtration barrier changes. It has been reported that anti‐dsDNA IgG binds to proximal renal tubular epithelial cells to promote inflammation and fibrosis (Taylor & Ryan, 2017; Tyagi, 2015). Consequential damage within the proximal tubule epithelium may impair albumin reabsorption (Venegas‐Pont et al., 2009) and account for the ineffectiveness of curcumin to lessen albuminuria at 34 weeks of age despite the attenuation of glomerular injury.

Plasma BUN levels and estimated GFR are used clinically to assess renal function (Momtazi‐Borojeni et al., 2018) and display an inverse relationship as increases in BUN and decreases in GFR have been reported in this patient population. BUN levels in SLE mice have been reported to be increased by ~32 weeks of age (Xu, Long, Dai, & Liu, 2007; Yao et al., 2016) and 31 weeks of age (Mollazadeh et al., 2019). The present study demonstrates curcumin‐treated SLE mice have lower plasma BUN levels at 34 weeks of age compared to age‐matched vehicle‐treated SLE mice. This was statistically evident at 34 weeks of age after 2 weeks of treatment, a time when renal injury has already been established in this model (Mollazadeh et al., 2019). While the curcumin‐treated SLE mice had significantly reduced levels of plasma BUN at 34 weeks of age, curcumin‐treated SLE mice at 28 weeks of age did not reach statistical significance when compared to age‐matched vehicle counterparts. This may be reflective of the biological variability common in this model that may be overcome with an increased sample size. Interestingly, curcumin‐treated SLE mice at 28 weeks of age seem to be protected from the increase in BUN that is already evident in some of the vehicle‐treated age‐matched SLE mice. Although SLE mice had lower GFR by 34 weeks of age, this was not restored in curcumin‐treated mice. Thus, the current study suggests that curcumin may have some beneficial effects to improve renal function.

The kidneys have a central role in the long‐term control of blood pressure, and the lower GFR with increased BUN in SLE mice are consistent with renal function changes that contribute to hypertension. Hypertension is prevalent among SLE patients and is a contributing factor to cardiovascular morbidity and mortality (Anders & Weening, 2013; Mayes et al., 2003; Yu, Gershwin, & Chang, 2014; Yung & Chan, 2012, 2015; Yung, Ng, et al., 2017), and is evident in female NZBWF1 SLE mice (Han et al., 2015; Yung, Yap, & Chan, 2017). The present study demonstrates curcumin treatment had no significant effect on MAP in control or SLE mice. Because blood pressure is not significantly altered in curcumin‐treated animals, it is unlikely that the modestly reduced renal injury is the result of changes in systemic hemodynamic function, but rather related to potential anti‐inflammatory properties of curcumin directly acting at the tissue level.

Curcumin is an affordable adjunctive therapeutic option for SLE mice with potential translational efficacy in humans as the current study has demonstrated that treatment with oral curcumin may decrease autoimmune activity (differences in spleen weight), renal injury (decline in glomerulosclerosis), and improve renal function (decreased BUN) in female NZBWF1 SLE mice. The lack of significant differences in circulating CD45R+ B cells, anti‐dsDNA IgG autoantibodies, albuminuria, GFR, and MAP in curcumin‐treated mice may be reflective of initiation time and duration of treatment. Further studies are needed to investigate timing and duration of therapy alongside commonly prescribed immunosuppressants such as mycophenolate mofetil to further investigate mechanisms of action on subsets of B‐ and T‐lymphocyte populations.

## DISCLOSURE

None.
